# Assignment of adverse event indexing terms in randomized clinical trials involving spinal manipulative therapy: an audit of records in MEDLINE and EMBASE databases

**DOI:** 10.1186/s12874-017-0320-x

**Published:** 2017-03-14

**Authors:** Lindsay M. Gorrell, Roger M. Engel, Reidar P. Lystad, Benjamin T. Brown

**Affiliations:** 1 Human Performance Laboratory, KNB 222, Faculty of Kinesiology, University of Calgary, Calgary, Alberta T2N 1 N4 Canada; 2 Department of Chiropractic, Macquarie University, Building C5C West, Sydney, 2109 Australia; 3 Australian Institute of Health Innovation, Faculty of Medicine and Health Sciences, Macquarie University, Level 6, 75 Talavera Road, Sydney, NSW 2109 Australia

**Keywords:** Indexing, Adverse events, Harms, Spinal manipulative therapy, Literature review

## Abstract

**Background:**

Reporting of adverse events in randomized clinical trials (RCTs) is encouraged by the authors of The Consolidated Standards of Reporting Trials (CONSORT) statement. With robust methodological design and adequate reporting, RCTs have the potential to provide useful evidence on the incidence of adverse events associated with spinal manipulative therapy (SMT). During a previous investigation, it became apparent that comprehensive search strategies combining text words with indexing terms was not sufficiently sensitive for retrieving records that were known to contain reports on adverse events. The aim of this analysis was to compare the proportion of articles containing data on adverse events associated with SMT that were indexed in MEDLINE and/or EMBASE and the proportion of those that included adverse event-related words in their title or abstract.

**Methods:**

A sample of 140 RCT articles previously identified as containing data on adverse events associated with SMT was used. Articles were checked to determine if: (1) they had been indexed with relevant terms describing adverse events in the MEDLINE and EMBASE databases; and (2) they mentioned adverse events (or any related terms) in the title or abstract.

**Results:**

Of the 140 papers, 91% were MEDLINE records, 85% were EMBASE records, 81% were found in both MEDLINE and EMBASE records, and 4% were not in either database. Only 19% mentioned adverse event-related text words in the title or abstract. There was no significant difference between MEDLINE and EMBASE records in the proportion of available papers (*p* = 0.078). Of the 113 papers that were found in both MEDLINE and EMBASE records, only 3% had adverse event-related indexing terms assigned to them in both databases, while 81% were not assigned an adverse event-related indexing term in either database.

**Conclusions:**

While there was effective indexing of RCTs involving SMT in the MEDLINE and EMBASE databases, there was a failure of allocation of adverse event indexing terms in both databases. We recommend the development of standardized definitions and reporting tools for adverse events associated with SMT. Adequate reporting of adverse events associated with SMT will facilitate accurate indexing of these types of manuscripts in the databases.

## Background

The purpose of a randomized clinical trial (RCT) is to collect and appropriately report both beneficial and harmful effects from an intervention and to compare these outcomes across groups. However, it is far more common for RCTs and in turn, systematic reviews, to focus on the beneficial effects of an intervention versus harms data when reporting results [1]. Recognition of this inequality in reporting is important as it may bias the ability of both health-care practitioners and patients to accurately assess the risk-benefit ratio of an intervention.

Adverse events which have been associated with spinal manipulative therapy (SMT) range from catastrophic, such as cervical artery dissection resulting in stroke to mild, transient muscle soreness or stiffness [2–4]. Typically, catastrophic events are rare while minor events are more common and are considered by some authors to be an expected treatment outcome [4, 5]. While maintaining a focus on catastrophic events is understandable [6–8], quantifying the incidence of other types of adverse events associated with SMT is also necessary in order to accurately inform prognoses, and management decisions [5, 9, 10]. Although previous reports on the incidence of minor and moderate adverse events following manual therapy [5] are welcome, their conclusions are limited as they were based on a small number of trials and it is not clear exactly how representative these reports are of the incidence of mild and moderate adverse events specifically associated with SMT [5, 11].

Adequate reporting of adverse events in RCTs is encouraged by the authors of The Consolidated Standards of Reporting Trials (CONSORT) statement which was first published in 1996 and subsequently updated in 2001 and 2010 respectively [12–14]. Furthermore, in 2004 an extension to the statement was published which attempted to directly address the reporting of adverse events. Specifically, this extension discussed the inadequacy of current adverse events reporting and provided 10 recommendations for reporting which were later adopted in the 2010 version of the statement [15]. With robust methodological design and adequate reporting, RCTs, in conjunction with case reports and observational studies, have the potential to provide useful evidence on the incidence of adverse events associated with SMT. Comprehensive reporting of adverse events (and exposures) across multiple trials could allow for pooled analyses over time and may provide a way of estimating the incidence rates for all classifications (minor – catastrophic) of adverse events thereby helping to inform management strategies.

During a previous investigation into the reporting of adverse events associated with SMT, it became apparent that comprehensive search strategies combining text words (e.g., “spinal manipulation” or “spinal manipulative therapy”) with indexing terms (e.g., “adverse event” or “adverse effect”) were not sufficiently sensitive for retrieving records that were known to contain reports on adverse events [16]. This observation was based on recent searches of the Central Register of Controlled Trials (CENTRAL) and Physiotherapy Evidence Database (PEDro) which index approximately 97% and 95% of all RCTs involving manual therapy respectively [17, 18].

Our primary objectives, therefore, were: (1) to determine the completeness of assignment of adverse event-related indexing terms in MEDLINE and EMBASE records of a pool of papers known to report on adverse events following SMT; (2) to compare the completeness of assignment of adverse event-related indexing terms in MEDLINE and EMBASE records; and (3) to determine the proportion of papers in which SMT was an intervention that included adverse event-related words in their title or abstract. While similar investigations have been performed to assess the adequacy of adverse event reporting in the literature relating to pharmaceutical [1, 19] and physiotherapy interventions [17], this is the first study to investigate the completeness of adverse event indexing in RCTs involving SMT.

## Methods

We used a reference sample of 140 RCT articles previously identified as containing data on adverse events associated with SMT [16]. These articles were identified using a comprehensive search that included electronic searching of PEDro and Cochrane CENTRAL from inception to February 2016 and subsequent snowballing strategies. See Appendix 1 for the complete search strategy for the Cochrane CENTRAL database.

Each article in the reference sample was manually checked to determine if: (1) it had been indexed with relevant terms describing adverse events in the MEDLINE and EMBASE databases; and (2) it mentioned adverse events (or any related terms) in the title or abstract (thus enabling the paper to be found in an electronic search using text words).

### Brief overview of EMBASE and MEDLINE indexing processes

For articles published in English, the indexing in EMBASE follows the Elsevier Life Science Thesaurus – Emtree. This thesaurus contains over 70,000 biomedical preferred terms and 290,000 synonyms that are ordered within 14 facets e.g., healthcare concepts. For the most part, indexing terms are manually chosen based on the full-text content of an article. This process is carried out by trained indexers with a biomedical background according to well-defined guidelines that are subject to regular quality assurance checks. The relevant concepts within an article are identified and matched with specific Emtree terms. Each indexing term is designated as either a major or minor term based on relevance to the concepts presented within each article. Articles are indexed with an average of 3–4 major terms and several minor terms, with major terms being weighted more heavily than the minor with regard to relevance during a search. With regards to harms- related indexing terms in EMBASE, the indexing framework has been built primarily around drug therapy with common terms including *side effect*, and *adverse drug reaction*. All adverse events/effects are indexed with the term *side effect* with no consideration given to the severity of the adverse event/effect. Terms that are not currently indexed on Emtree are indexed as *candidate* terms. These candidate terms are evaluated regularly for possible inclusion on the Emtree thesaurus [20].

The indexing process is similar in MEDLINE. Indexers screen the title, abstract, body and bibliography of the article for relevant concepts and compare these with the keywords suggested by the author/publisher. The indexers then use the National Library of Medicine *MeSH Browser* to find indexing terms (*major* and *non-major*) that most accurately match the concept being indexed from the Medical Subject Heading (MeSH) thesaurus [21]. The MeSH vocabulary is continually revised and updated based on suggestions from indexers, and consultation with professionals from various disciplines with specialized vocabularies [22]. The headings relating to harms in the MeSH database include the following: *patient harm*, *drug-related side effects and adverse reactions*, *drug eruptions*, *iatrogenic disease*, and *long term adverse effects* [23].

### Details of papers included in the review

Spinal manipulative therapy was defined as manual therapy involving a high-velocity, low amplitude manipulation directed at a spinal joint with the intention of moving the joint past its physiological range of motion without exceeding the anatomical limit [24, 25]. Spinal manipulation delivered using mechanical instruments and drop-table mechanisms were also included in this review as they have been classified as high-velocity, low amplitude procedures in the literature [24, 26].

Randomized clinical trials that reported original data from SMT, either as the sole intervention or as part of a multi-modal intervention, delivered by a regulated manual therapy practitioner were eligible for inclusion. We excluded manuscripts reporting all other trial designs, commentaries, editorials, reviews, trial registrations and protocols, conference proceedings, full text articles not available in English, articles that had been retracted, secondary analyses, articles reporting on interventions that were applied to regions other than the spine, if the intervention was self-administered (e.g., exercise), and finally if it was unclear whether or not the SMT applied was high-velocity and low amplitude in nature. The eligibility criteria were applied at each stage of the selection process. An assessment of the risk of bias was performed for all included articles using the Cochrane risk of bias assessment tool [27].

### Data analysis

The completeness of records in MEDLINE and EMBASE was determined by calculating proportions, which were subsequently compared using an exact McNemar test with a significance level set at 0.05. The completeness of assignment of adverse event-related indexing terms to database records and comparison of proportions were determined in the same manner. The proportion of papers using adverse event-related indexing terms in the title or abstract was calculated, and the frequencies of adverse event-related indexing terms used in titles or abstracts, MEDLINE records, and EMBASE records were collated separately. All analyses were conducted using the statistical software package R, version 3.3.1 (R Core Team, Vienna, Austria).

## Results

Of the total pool of 140 papers, 128 (91%) and 119 (85%) papers had records in MEDLINE and EMBASE, respectively. While 113 (81%) papers had records in both MEDLINE and EMBASE, 6 (4%) papers did not have records in either database. There was no statistically significant difference between MEDLINE and EMBASE in the proportion of available records (*p* = 0.078). Table 1 provides an overview of the frequency and proportion of records in MEDLINE and EMBASE.

**Table 1:** Frequency and proportion of available papers (*n* = 140) with records in MEDLINE and EMBASE

Database	Number of papers	Proportion of available papers (95% CI)
MEDLINE	128	91% (86–95%)
EMBASE	119	85% (78–90%)

Of the 113 papers that had records in both MEDLINE and EMBASE, 13 (12%) and 12 (11%) papers were assigned an adverse event-related indexing term in MEDLINE and EMBASE, respectively. Only 3 (3%) papers had adverse event-related indexing terms assigned to them in both databases, while 91 (81%) papers were not assigned an adverse event-related indexing term in either database. There was no difference between MEDLINE and EMBASE in the proportion of records being assigned an adverse event-related indexing term (*p* = 1.000). Table 2 provides an overview of the frequency and proportion of records that were assigned one or more adverse event-related indexing terms.

**Table 2:** Frequency and proportion of papers with records in both MEDLINE and EMBASE (*n* = 113) that were assigned one or more adverse event-related indexing term

Database	Number of papers	Proportion of available papers (95% CI)
MEDLINE	13	12% (6–19%)
EMBASE	12	11% (6–18%)

Of the total pool of 140 papers, only 27 (19%) of papers mentioned adverse event-related terms in the title or abstract. See Table 3 for a detailed overview of the frequency of adverse event terms used in titles or abstracts, as well as in MEDLINE and EMBASE records.

**Table 3:** Frequency of adverse event-related terms used in titles or abstracts, MEDLINE records, and EMBASE records

Title or abstract	MEDLINE	EMBASE
Adverse events (*n* = 14)	Modality^a^/adverse effects (*n* = 16)	[Side Effect] (*n* = 5)
Side effects (*n* = 6)	Symptom^a^/complications (*n* = 1)	[Adverse Drug Reaction] (*n* = 6)
Adverse reactions (*n* = 4)	Risk Assessment (*n* = 2)	Symptom^a^/si [Side Effect] (*n* = 4)
Adverse effects (*n* = 3)	Risk Factors (*n* = 1)	Symptom^a^/ae [Adverse Drug Reaction] (*n* = 2)
Harm (n = 1)		Symptom^a^/co [Complication] (*n* = 48)
Unpleasant reaction (*n* = 1)		Drug effect (*n* = 1)
Discomfort (*n* = 1)		
Sore (*n* = 1)		
Serious complication (*n* = 1)		
Side effects (*n* = 6)		
Adverse effects (*n* = 3)		

^a^Any specifically named modality (e.g., spinal manipulative therapy) or symptom (e.g., neck pain)

## Discussion

The objective of this study was to investigate the completeness of indexing of adverse events in RCTs involving SMT in the MEDLINE and EMBASE databases. To the authors’ best knowledge, this is the first review of its kind in the SMT literature. Our results show that while there was effective indexing of the manuscripts in the databases, which is consistent with the existing literature [17, 18], there was inadequate allocation of adverse event indexing terms in each database. Specifically, that 81% of papers did not have an adverse event-related indexing term assigned to them is unacceptable.

There are a number of reasons for this short-coming. Firstly, there may have been a failure of authors to capture and report on adverse events during the RCT. Well-designed RCTs are the gold standard for investigating the efficacy of healthcare interventions and ultimately provide the most reliable results [28, 29]. This outcome is only achieved when there are adequate data collection and reporting processes for both the beneficial and harmful effects of an intervention. [30]. Neglecting to collect and report on adverse events is a failure to adequately investigate an intervention. Secondly, if adverse events data were collected, they may not have been adequately or appropriately reported. Examples of inadequate reporting include failure to fully report the nature and timing of events or the number of interventions (manipulations) provided in a trial which subsequently precludes calculation of the incidence rate for an event. Inappropriate reporting includes omitting the results from the article abstract. After the title, the abstract is the second most read section of a scientific paper [31]. Our analysis shows that reporting of adverse events associated with SMT in RCTs is inadequate and that very few authors include this information in the abstract. Although indexing terms are allocated by trained indexers with a biomedical background and are based on the full-text content of an article, reporting on adverse events data in a key position such as the abstract will undoubtedly make the assignment of indexing terms less difficult for database curators [16].

The inadequate reporting of adverse events is not unique to manual therapy. A review of seven different medical disciplines concluded that there was both a failure to distinguish between the severity of adverse events and that the overall reporting of events was inadequate [32], while others have highlighted the heterogeneity in the reporting of adverse events [33]. An evaluation of safety reporting in 15 RCTs involving complementary and alternative medicine reported inadequate reporting and emphasized that pooling of adverse events data was compromised as a result [34].

Finally, it is possible that due to a lack of standardized definitions and reporting tools, there may be confusion amongst authors as to what exactly constitutes an adverse event. What may be considered a mild adverse event (e.g., transient muscle soreness following a manipulation) by one author could be considered to be a normal effect of treatment by another and therefore not reported [4, 5]. Despite this, as discussed previously, the CONSORT guidelines [13] provide authors with a clear scaffold with which to report adverse events and as such, there is no excuse for poor reporting in RCTs. Comprehensive reporting of adverse events associated with SMT in RCTs that follow the CONSORT guidelines would no doubt facilitate adequate indexing of the manuscript in databases. Furthermore, some of the responsibility for the current inadequate level of adverse events reporting also resides with journal editors. Although many of the articles in this analysis were published in journals purportedly following the International Committee of Medical Journal Editors (ICJME) recommendations, [35, 36] in addition to those endorsing the CONSORT statement [13], there remains a failure of authors to adequately report both the beneficial and harmful effects of an intervention. To address this shortcoming, we advocate that journal editors ensure authors comply with CONSORT guidelines. One way to achieve this could be requesting that authors append a completed CONSORT checklist to their submission in addition to instructing peer reviewers to check that all items on the checklist are adequately addressed. Furthermore, journal editors need to ensure that authors are instructed to revise manuscripts with inadequate reporting of adverse events.

Adverse event-related indexing terms were reported in approximately 10% of all articles. There was considerable heterogeneity among the indexing terms used as seen in Table 3. In the MEDLINE database, the majority of indexing terms reported ‘Modality*/adverse effects’ while terms indicating ‘risk’ were also used. In the EMBASE database, a large number of the adverse events reported on the effects of drugs rather than SMT but were included in this analysis for completeness. Terms indicating ‘side effect’ were also commonly used.

The text words used to describe adverse events in the titles and abstracts of the articles also displayed marked heterogeneity. While we found that the term ‘adverse event’ was most frequently used, words such as ‘side-effects’, ‘adverse reactions’ and ‘effects’, ‘harms’ and ‘unpleasant reaction’ were also used. Notably, the definitions advocated by the CONSORT guidelines recommend the use of the term ‘harms’ which was only reported once in our analysis.

A ‘harm’ is defined as the totality of possible adverse consequences of an intervention or therapy; it is the direct opposite of benefit, against which it is being compared [15]. Thus, its use is recommended over terms such as ‘side effect’ which have traditionally been used to describe unintended drug effects and do not delineate between harmful or beneficial effects. In addition to this, it has the potential to understate the importance of harms as ‘side’ may be perceived as denoting secondary importance [37]. Furthermore, the term ‘side effect’ may be misleading as it suggests causality i.e., the effect is caused by the intervention being tested. In a typical RCT it may not be possible to ascertain whether an observed event is partially, entirely or not at all due to the intervention as there may be unknown, underlying and complicating comorbidities present. As mentioned previously, the purpose of an RCT is to collect and appropriately report both beneficial and harmful effects coinciding with the application of an intervention and to compare these outcomes across groups. The term ‘adverse event’ is therefore preferred when describing a harmful event that occurs during a trial.

As discussed above, lack of reporting on adverse events in the title and abstracts of reports on SMT obstructs the allocation of appropriate indexing terms, thus impeding research into adverse events in this area. Without information concerning the risks of SMT, consumers and practitioners alike do not have access to the information necessary to accurately inform a balanced risk-benefit analysis of this type of intervention [38, 39].

We advocate for a standardized definition for all classifications of adverse events and subsequent reporting tools specific to SMT. Furthermore, we recommend that journal editors enforce use of the CONSORT statement and thus adverse events reporting in the abstract. This would facilitate consistency of adverse event-related indexing terms in the future.

### Limitations

There are several limitations to this secondary analysis. Firstly, electronic searches for the original dataset were performed in two databases only (PEDro and Cochrane CENTRAL), which may have resulted in the omission of eligible articles found in other databases. However, we believe this is unlikely as both PEDro and Cochrane CENTRAL index RCTs published in all journals and Cochrane CENTRAL also indexes the grey literature. In addition to this, it has been reported that PEDro and Cochrane CENTRAL indexed 99% and 98% of 281 RCTs, respectively, when compared to PubMed, EMBASE and CINAHL [17]. Furthermore, our results concerning the indexing of the articles were similar to those results already published [17, 18].

Secondly, the review only included articles published in English. It has been reported that journals published in English are more likely to report positive findings [40]. It is uncertain what effect this language-bias may have had, but it is unlikely that the results would have been altered in any meaningful way as only 3 articles were excluded for this reason [41, 42].

Thirdly, we do not believe there is sufficient evidence to warrant an analysis of whether the indexing of adverse events associated with SMT in RCT has changed over time. It is possible that there has been an improvement in indexing resulting from the introduction of the CONSORT guidelines [43] and widespread acceptance of the ICMJE recommendations [36]. However, we do not consider this a major limitation as the paucity of adequate indexing outweighs any improvements which may have occurred.

Finally, for the sake of completeness, we included all indexing terms and text words indicating adverse events associated with RCTs, rather than focusing only on those related to SMT. This inclusion may have resulted in non-specific reporting of adverse events-related indexing terms which may have bolstered our interpretation of the appropriateness of such indexing.

## Conclusions

Despite effective indexing of RCTs involving SMT in the MEDLINE and EMBASE databases, there is a failure of allocation of adverse event indexing terms in both databases. This is likely due to a number of factors, specifically – a lack of standardized nomenclature surrounding adverse events associated with SMT, the inadequate reporting of adverse events in RCTs that involve SMT and a lack of understanding on behalf of authors about the mechanics of manuscript indexing in databases. We recommend the development of standardized definitions for adverse events associated with SMT, in addition to the development of standardized adverse events reporting tools which will in turn improve the reporting of adverse events by authors. Adequate reporting of adverse events associated with SMT will facilitate accurate indexing of these types of manuscripts in the databases. Finally, we suggest that education of manual therapy researchers and authors concerning the mechanics of manuscript indexing in databases be prioritized.

## Appendix 1

### Cochrane CENTRAL search string

#1 “spine”:ti, ab, kw or “spinal”:ti, ab, kw

#2 “manip*”

#3 MeSH descriptor: [Musculoskeletal, Manipulations] explode all trees

#4 MeSH descriptor: [Manipulation, Spinal] explode all trees

#5 MeSH descriptor: [Manipulation, Chiropractic] explode all trees

#6 MeSH descriptor: [Manipulation, Osteopathic] explode all trees

#7 “osteopath*”

#8 “chiropract*”

#9 #1 and #2

#10 #3 or #4 or #5 or #6 or #7 or #8 or #9

## Abbreviations

CENTRAL: Central Register of Controlled Trials
CONSORT: Consolidated Standards of Reporting Trials
ICMJE: International Committee of Medical Journal Editors
MeSH: Medical subject headings
PEDro: Physiotherapy Evidence Database
RCT: Randomized clinical trial
SMT: Spinal manipulative therapy
